# Medical Morbid Anatomy and Pathology

**Published:** 1910-06

**Authors:** 


**Medical Morbid Anatomy and Pathology.** By H. Thursfield, M.D., F.R.C.P., and W. P. S. Branson, M.D., M.R.C.P Pp. viii, 262. London: H. K. Lewis. 1909.

A most worthy book which, masquerading as a museum catalogue, turns out on closer acquaintance to be a series of essays on morbid anatomy, written in crisp, vigorous, and intelligible English. (It is so long since we met our mother tongue in a medical treatise.) The descriptions of the results of disease in the various organs are excellent, though room might have been found for the more recent observations on myocardial degenerations and heart rhythm. Syphilis as manifested in the mouth is not very adequately set forth: primary chancres are said to be "found most often on the lips or tongue," although the majority of observers regard tonsillar chancre as the commonest extra-genital primary lesion. The characters of the secondary "snail-crawl" ulceration are not made plain, and Hutchinson's teeth are described as "wider at the margins than the base," which, if by margin is meant the cutting edge, is certainly inaccurate.

Many tropical infections, such as sprue and kala-azar, now becoming familiar in England, go unnoticed. The French observations on sporotrichosis and allied mycoses are also disregarded. And surely abdominal aneurysm has a medical aspect.

We point to these omissions not in a captious spirit, but because the work is so excellent that a second edition is inevitable ; and there, perhaps, brief reference will be made to alien, but no longer unfamiliar, diseases.

The format of the volume is attractive, and typographical errors are few (*e.g.* "carnfication" on p. 103, and "splenculi" on p. 201) ; but why are we allowed no diphthongs in such words as amœbæ ?

The book is one which every student and practiser of physic will read with advantage and delight, while those who appreciate the literary flavour of a scientific repast will return with added zest to read again.

